# The Protective Effect of Antioxidants Consumption on Diabetes and Vascular Complications

**DOI:** 10.3390/diseases4030024

**Published:** 2016-07-11

**Authors:** Stéphanie Dal, Séverine Sigrist

**Affiliations:** DIATHEC EA 7294 UMR Centre Européen d’Etude du Diabète (CeeD), Université de Strasbourg (UdS), boulevard René Leriche, Strasbourg 67200, France; s.dal@ceed-diabete.org

**Keywords:** diabetes, complications, oxidative stress, antioxidants, plants, prevention

## Abstract

Obesity and diabetes is generally accompanied by a chronic state of oxidative stress, disequilibrium in the redox balance, implicated in the development and progression of complications such as micro- and macro-angiopathies. Disorders in the inner layer of blood vessels, the endothelium, play an early and critical role in the development of these complications. Blunted endothelium-dependent relaxation and/or contractions are quietly associated to oxidative stress. Thus, preserving endothelial function and oxidative stress seems to be an optimization strategy in the prevention of vascular complications associated with diabetes. Diet is a major lifestyle factor that can greatly influence the incidence and the progression of type 2 diabetes and cardiovascular complications. The notion that foods not only provide basic nutrition but can also prevent diseases and ensure good health and longevity is now attained greater prominence. Some dietary and lifestyle modifications associated to antioxidative supply could be an effective prophylactic means to fight against oxidative stress in diabesity and complications. A significant benefit of phytochemicals (polyphenols in wine, grape, teas), vitamins (ascorbate, tocopherol), minerals (selenium, magnesium), and fruits and vegetables in foods is thought to be capable of scavenging free radicals, lowering the incidence of chronic diseases. In this review, we discuss the role of oxidative stress in diabetes and complications, highlight the endothelial dysfunction, and examine the impact of antioxidant foods, plants, fruits, and vegetables, currently used medication with antioxidant properties, in relation to the development and progression of diabetes and cardiovascular complications.

## 1. Introduction

Today, WHO and IDF (*International Diabetes Federation*) draws attention to the similarity of trends in obesity and diabetes in the World. The term “diabesity” is commonly used today to describe this epidemic or pandemic with exponential dramatic growth observed in all countries [1]. Our change of lifestyle to a sedentary attitude and massive industrialization with access from an early age to food and beverages rich in energy, fat, sugar, or a combination thereof is partly the cause of millions of obese and diabetic people [2]. Despite technical and technological progress accompanying therapeutic arsenal available and public health plans, we fail today to stop the progression of diabetes and its complications. In fact, diabetes is a silent and sneaky disease. Therefore, it is associated with many complications. Cardiovascular diseases are the major cause of death and disability among diabetic people [3], particularly for woman who have lost cardiovascular protection afforded by the classically female sex. Diabetic vascular complications are an important pathological issue in diabetes that leads to the further functional deterioration of several organs and caused micro- and macro-angiopathy [4]. Endothelial dysfunction, the loss of a balance between vasodilators and vasoconstrictors factors in the blood vessels, has largely been associated in several regions of the vasculature in T2D [5].

A common point of all these cardio-metabolic disorders is the appearance of oxidative stress. Oxidative stress is due to an imbalance between antioxidants (enzymes, vitamins, proteins, etc.) and pro-oxidants (UV radiations, alcohol, smoking, etc.) [6]. Oxidative stress along with chronic low-grade inflammation may initiate changes in cardiovascular structure and function such as endothelial dysfunction, cardiac hypertrophy, cardiac fibrosis, and ventricular contractile dysfunction [7]. Many studies have shown that diabetic patients undergo chronic oxidative stress, particularly due to hyperglycemia [8,9]. Thus, a strategy focus on both oxidative stress and endothelial function could help to prevent or delay the onset of vascular-related type 2 diabetes complications.

Much evidence shows that consumption of natural source substances confers chemopreventive and cytoprotectant activities. In fact, epidemiological studies suggest that consumption of fruits, vegetables [10,11,12,13,14], and plants [15] may be associated with a reduced risk of diabetes or a protective effect [16]. Some observations have also revealed an inverse relationship between the risk of cardiovascular mortality or morbidity and the consumption of polyphenol-rich products (red wine, cocoa and tea) [17,18,19,20]. Their consumption brings several exogenous antioxidants and vitamins, increasing the antioxidant status of the organism, in addition to their direct effect on blood vessels and in particular on the endothelium [21].

Many plants are also used for their benefits in traditional medicines. Some of them are at the origin of the development of drugs [16] such as biguanide, metformin, antidiabetic drugs, and *Galega officinalis*. In developed countries, traditional, complementary, and alternative medicines are becoming increasingly popular and are commonly used to treat or prevent chronic diseases and are improving quality of life [15].

Therefore, we will see through this review that many compounds surrounding us can be a real asset in the prevention of “diabesity” but also a valuable aid in addition to current treatments to prevent the occurrence of such complications. We will also discuss the appeal for the use of single molecules to the detriment of total extracts, thereby promoting molecular synergy.

## 2. Diabesity and Cardiovascular Complications

### 2.1. The Evolution of Obesity and Diabetes

Developed societies face two crucial health problems: overweight and obesity. Obesity is the most common metabolic disease, and the number of individuals who are overweight or obese is fast increasing worldwide [22]. Overweight and obesity are defined as abnormal or excessive fat accumulation that may impair health. Body mass index (BMI) is a simple index of weight-for-height that is commonly used to classify overweight and obesity in adults. It is defined as a person's weight in kilograms divided by the square of his height in meters (kg/m^2^). BMI is widely used as a measure of weight status and disease risk and is widely used for routine characterization of weight status in epidemiology, clinical nutrition, and research. Moreover, fat mass and fat-free mass as assessed by validated techniques (densitometry, dual impedance analysis, etc.) are also currently used. Thus, the term obesity in our review is used in a broad sense that includes BMI, fat mass, % body fat, etc. 

Obesity has more than doubled since 1980. The prevalence of overweight and obese youth has increased dramatically over the past three decades [23]. In 2014, more than 1.9 billion adults, 18 years and older, were overweight. Of these over 600 million were obese. 39% of adults aged 18 years and over were overweight in 2014, and 13% were obese. Most of the world’s population lives in countries where overweight and obesity kills more people than does underweight.

Overweight and obesity have reached epidemic proportions globally along with an adoption of a Westernized lifestyle characterized by a combination of excessive food intake and inadequate physical activity. Raised BMI is a major risk factor for noncommunicable diseases such as cardiovascular diseases (mainly heart disease and stroke), which were the leading cause of death.

The dramatic rise in the prevalence of obesity and changes in lifestyle-related factors such as a reduction in physical activity have been accompanied by alarming increases in the incidence and prevalence of type 2 diabetes [24]. Diabetes is a chronic disease that occurs either when the pancreas does not produce enough insulin or when the body cannot effectively use the insulin it produces. Insulin is a hormone that regulates blood sugar [25]. Hyperglycemia, or raised blood sugar, is a common effect of uncontrolled diabetes and, over time, leads to serious damage to many of the body's systems, especially the nerves and blood vessels. In 2014, 9% of adults 18 years and older had diabetes. In 2012, diabetes was the direct cause of 1.5 million deaths. More than 80% of diabetes deaths occur in low- and middle-income countries.

Epidemiological studies have confirmed a strong positive association between excess adiposity and risk of developing type 2 diabetes. Based on the data from the Behavioral Risk Factors Surveillance System conducted by the United States, Mokdad et al. [26] estimated that, for every kilogram increment in self-reported body weight, the risk of diabetes increases by about 9%. The term “diabesity” has been coined to illustrate the close relationship between obesity and diabetes [27,28].

### 2.2. Lifestyle

Lifestyle habits have deteriorated over time with increases in obesity, central obesity, and diabetes and stagnating rates of persistent smoking. An increase in obesity and diabetes has paralleled the growth of urbanization and globalization in the region. For example, in China, the prevalence rates of diabetes in large provincial capital cities range from a high of 8% (in the Eastern region) to 4.6% (in the lowest in the Western region) [29]. Behavioral risk factors include tobacco use, alcohol consumption, unhealthy diet, and physical inactivity. Physical inactivity is the 4th mortality risk factor for mortality [30] with an increase of 20–30% of death compared with people who practice 30 min of exercise a day [31].

Finally, advances in agriculture and food systems, consequent increases in food availability, and a shift in dietary consumption patterns with economic development and urbanization of developing societies promotes overweight and obesity. This “new” diet favors consumption of fats, saturated fats largely from animal sources and sugars. The essence of these changes is captured by the term “nutrition transition” which accompanies the demographic and epidemiologic transition in these countries with economic development [32].

Epidemiological studies indicate that weight loss, even moderate, can improve insulin sensitivity, improve insulin action, and decrease the risk of developing type 2 diabetes. Improvements in insulin action after an average of 10% weight reduction were lost with weight regain but largely preserved with weight maintenance [33].

Physical activity is associated with a significant reduction in the risk of type 2 diabetes, whereas a sedentary lifestyle is associated with an increased risk [34,35]. There is a 20% increased risk of diabetes for each 2-h daily increment in watching television [36]. However, some studies have demonstrated the feasibility and efficiency of lifestyle intervention programs in the prevention of diabetes in individuals with impaired glucose tolerance [37,38]. The lifestyle intervention program permits a reduction in weight with moderate exercise and a controlled food intake (reduction of fat, increase in fiber, and frequent consumption of fruits, vegetables, etc.)

### 2.3. Diabetic Complications: Link with Oxidative Stress and Inflammation

Chronic hyperglycemia, disturbances of carbohydrate, and lipid and protein metabolism lead to metabolic derangements in diabetes and various complications including both macro- and microvascular dysfunctions [22]. Over time, diabetes can damage the heart, blood vessels, eyes, kidneys, and nerves. The incidence of cardiovascular diseases in people with diabetes, one of the major complications, is three to four times that in non-diabetic individuals. In a multinational study, 50% of people with diabetes die of cardiovascular disease (primarily heart disease and stroke) [39], with a twofold increase in risk of heart failure in male patients, and a fivefold increase in female patients [40]. Likewise, diabetes increased incidence of coronary artery disease and atherosclerotic lesions at a younger age, often associated with multivessel disease and involvement of distal coronary segments. Hypertension is also commonly found in both type 1 and type 2 diabetes [41]. Finally, diabetes can cause distinct pathologic alterations in the myocardium, independent of its effect on blood pressure and coronary atherosclerosis, termed “diabetic cardiomyopathy” (DMC) [42]. We will focus on cardiovascular complications below in this review.

Combined with reduced blood flow, neuropathy (nerve damage) in the feet increases the chance of foot ulcers, infection, and the eventual need for limb amputation and affects almost 30% to 50% of patients with diabetes. One percent of global blindness is attributed to diabetic retinopathy [43] due to a long-term accumulated damage to the small blood vessels in the retina, and the overall risk of dying among people with diabetes is at least double the risk of their peers without diabetes [44]. Another important microvascular complications is diabetic nephropathy, of which there is a ninefold higher risk in patients with diabetes, leading to end-stage renal disease requiring chronic dialysis and transplantation [23].

Oxidative stress has been suggested to be a common pathway for the pathogenesis of complications in diabetes [24,25]. For example, (1) the production of hydrogen peroxide by mesangial cells and lipid peroxidation, activation of protein kinase C (PKC), mitogen-activated protein (MAP) kinases, and cytokine production lead to renal injury [26]; (2) the redox-sensitive nuclear transcriptional factor, NFκB, accumulation of advanced-glycation end-products (AGEs) localized in sub-retinal membranes, and microvessels are activated earlier in the course of diabetic retinopathy [23,27] in addition to polyol accumulation and glycation associated to cataract [28]; (3) AGEs inhibit axonal regeneration [29], an increase in DNA damage and the stimulation of the PKC pathway, and NFκB and TGF-β increase deposition of the extracellular matrix [30], and all mechanisms have involved in neuropathy. Moreover, HbA1c, a biomarker of the overall glycemic exposure, is the most known diabetic parameter link to oxidative stress. In fact, it is due to the glycation of hemoglobin. The increase in Hb1Ac variability predicts the risk of microvascular complications in T1D [31,32,33] and the risk of nephropathy and cardiovascular diseases in T2D [34,35,36].

In addition to oxidative stress, inflammation stands out as a determinant process in the development of diabetic complications [37]. It is difficult, in fact, to understand the impact of these factors without each other, since numerous interplays exist between inflammation and oxidative stress and vice versa [38,39]. Hyperglycemia increases the levels of pro-inflammatory proteins [37], and infiltrated macrophages secrete inflammatory cytokines (correlate with a higher body mass index: IL-6, IL-8, MCP-1 [43]), thereby leading to a local and systemic inflammation. Increased production of TNF-α has also been widely associated with obesity-related insulin resistance and abnormal vascular reactivity, the vasculature being an important target of TNF-α [44] and closely linked to diabetic micro- and macro-complications [40,45].

## 3. Oxidative Stress and Cardiovascular Complications

**Figure 1:**
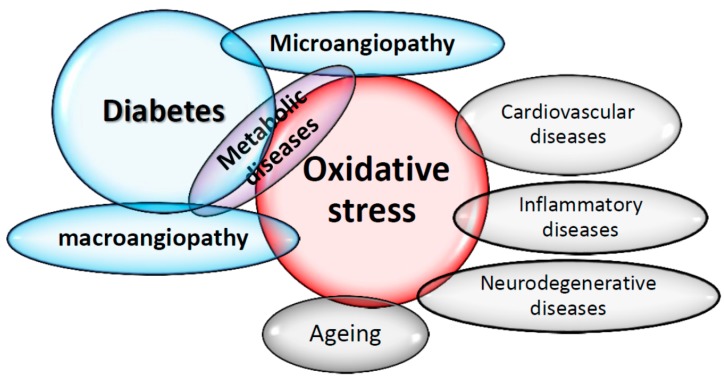
Oxidative stress in the middle of diseases and complications, including diabetes.

The concept that oxygen, which is essential to life, could be causing cell damage and involved in many diseases, was discovered in recent years. Today, many epidemiological and clinical studies strongly suggest the involvement of reactive oxygen species (ROS) in the genesis and evolution of chronic diseases, including diabetes and its complications [7] (Figure 1). Chronic hyperglycemia caused a major oxidative stress [22], and Yubero-Serrano et al. [46] recently proposed SOD activity as the most relevant oxidative stress biomarker in patients suffering from metabolic syndrome. It could be used as a predictive tool to determine the degree of the underlying oxidative stress in this pathology.

### 3.1. Oxidative Stress: A Question of Balance

#### 3.1.1. Oxygen Paradox and Anti-Oxygen

Oxygen, which first appeared three billion years ago in Earth's atmosphere, is an essential molecule for life. Through redox mechanisms, oxygen, the final electron acceptor, is transformed into water by the mitochondrial respiratory chain [41]. This reaction is a source of energy through ATP production and also the formation of 2% to 3% of reactive oxygen species (ROS), a free radical that is particularly unstable and reactive [42]. In 1954, Gerschman published the free radical theory of oxygen toxicity, due to partially reduced forms of oxygen [47], and, two years after, Harman proposed the concept of involving free radicals in the aging process [48]. Whereas McCord and Fridovich discovered the enzyme superoxide dismutase (SOD) in 1969 [49] and provided convincing evidence about the importance of free radicals in the living system [50], the concept of anti-oxidants has been reported for much longer by Dufraisse and Moureu in the 1920s, when they discovered that the polymerization of acrolein was inhibited by hydroquinone, an oxygen-dependent mechanism [51]. Originally named “anti-oxygen,” the Anglo-Saxon term “antioxidant” was quickly privileged and replaced. Since the properties as second messengers of ROS were discovered for the first time by Mittal and Murad in 1977 [52], many studies are now interested in this delicate balance between the beneficial and harmful effects of free radicals, which is the redox regulation for maintaining redox homeostasis and has provided protection to living organisms from various oxidative stresses.

#### 3.1.2. Free Radicals, Oxidative Stress, and Diabetes

**Figure 2:**
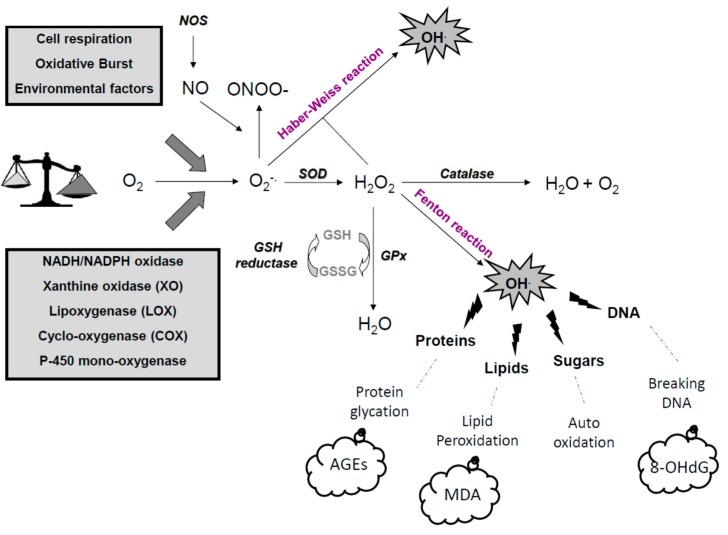
Oxidative defense and complications. AGEs: advanced glycated end-products; COX: cyclooxygenases; H_2_O_2_: hydrogen peroxide; LOX: lipoxygenases; NO: nitric oxide; NOS: NO synthase; NADPH oxydase: nicotinamide adenine dinucleotide oxidase; MDA: malondialdehyde (lipid peroxidation); SOD: superoxyde dismutases; GPx: glutathione peroxydase; GSH gluthathione; O_2_^.−^:superoxide anion; ONOO^.−^: peroxynitrite; OH. hydroxyl radical; 8-OHdG: 8-hydroxy-2’-deoxyguanosine (DNA damages).

Beside physiological oxidations, many environmental processes have induced free radical formations: air pollutants [53], tobacco [54], UV radiation from sun [55], and industrialized lifestyle [56]. Different endogenous enzymes can also form free radicals at physiological concentrations: NADPH oxidase, xanthine oxidase, cyclo-oxygenases (COXs), and lipo-oxygenases (LPOs), nitric-oxide synthases (NOS), P450 cytochrome, and mitochondrial chain [57]. These free radical were reduced by the first line of antioxidant defense: the superoxide dismutase SOD [58]. Free radicals include reactive nitrogen species (RNS) and reactive oxygen species (ROS). The most important is superoxide anion (O_2_^.−^), which is rapidly dismutated into oxygen and hydrogen peroxide (H_2_O_2_) by superoxide dismutase (SOD). Then, catalase (CAT) dismutates H_2_O_2_ into water and oxygen, and glutathione peroxidase (GPx) reduces both H_2_O_2_ and organics hydroperoxides (ROOH). However, in the presence of transition metals such as iron or copper, O_2_^.−^ and H_2_O_2_ form the strong oxidant hydroxyl radical (OH^.^) via Fenton reaction and the Haber–Weiss reaction. With chloride ions and H_2_O_2_, myeloperoxidase produce hypochlorous acid (HOCl). Nitric oxide (NO^.^) is produced from oxygen by various nitric oxide synthases (NOS) and produces the strong oxidant peroxynitrite (ONOO-) by reacting with O_2_^.−^. No enzymatic process can degrade ONOO–; however, with the presence of CO_2_, it form nitrate anion (NO_3_^−^) and nitrogen dioxide (NO_2_) (Figure 2).

In diabetes, the alteration of the first sites in the mitochondrial membrane lead to the activation of the complex II [59] and contribute to the formation of excessive O_2_^.−^ by a leakage of electrons [60]. NADPH oxidases (Nox’s), a family of enzymes with the sole function of producing ROS, are implicated in the pathophysiology of many cardiovascular diseases [61,62,63,64] and are the major source of glucose-induced ROS production in the vasculature [65,66], kidney [65], liver [66,67], and β cells [68], confirming this enzyme as a mediator of diabetic complications. Recently, Brandes et al. [69] described molecular mechanisms of Nox activation and supported their implications in diabetes, hyperglycemia, and hyperinsulinemia through complex pathways involving NADPH oxidases. Xanthine oxidase is also implicated in diabetes and vascular complications [70], whereas treatment of T2D patients with Allopurinol, a XO inhibitor, reduces the level of oxidized lipids in plasma and improves blood flow [70]. Glucose itself, as well as its metabolites, is known to react with hydrogen peroxide in the presence of iron and copper ions to form hydroxyl radical during auto-oxidation, described in diabetes and complications by Wolff and Dean in 1987 (Figure 2).

#### 3.1.3. Antioxidants Defenses

**Figure 3:**
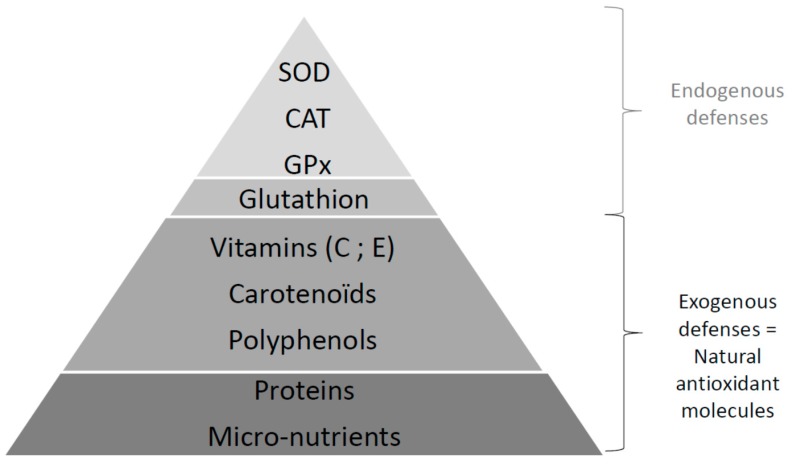
Oxidative defense strategies. CAT: catalase; GPx: glutathione peroxydase; SOD: superoxyde dismutases.

The body has a number of very effective antioxidant defense systems to lower the concentration of free radicals in the body. The term antioxidant refers to “any substance that, when present at low concentration compared with that of an oxidizable substrate, significantly delays or inhibits oxidation of the substrate” [71]. Nature of the antioxidant systems differs depending on the cell types, tissues, and localization in the intracellular or extracellular medium [72]. There are different types of molecules, natural or synthetic, with enzymatic or scavenging activities (Figure 3).

The first line of defenses against free radicals groups these enzymatic systems (SOD, CAT, GPx) (Figure 2 and Figure 3) and are aided by micronutrients (copper, zinc, selenium) [73] as cofactors. There are three isoforms for the SOD described in mammals [74]: the manganese-SOD (MnSOD) in the mitochondria, copper (Cu), or zinc (Zn) in the cytoplasm and the mitochondria, and both Cu/Zn extracellular SOD (Cu/Zn SOD) in vessels. CAT is essentially present in peroxisomes and in erythrocytes [75]. GPx is present in the extracellular fluid (blood) and in the cytoplasm and membranes of cells [76] and forms a couple with glutathione reductase (GR) providing glutathione (GSH) bioavailability [7].

The second line of defenses involves non-enzymatic antioxidants, such as naturally nutrients provided by food, with a scavenging effect (capture of free electron and formation of more stable entities), a stimulatory effect on endogenous antioxidant enzymes, or both [77]. Main molecules are GSH, vitamin E (the most active form: α-tocopherol), vitamin C (L-ascorbic acid), vitamin A (carotenoids), but also polyunsaturated fatty acids or exogenous flavonoids (quercetin, rutin, resveratrol, etc.), which can strengthen the antioxidant defenses of the body [73]. For example, increasing concentration of GSH with these products can protect against cancer [78] and diabetic complications [79]. Vitamin E traps organic free radicals from the oxidation of lipids and helps reduce lipid peroxidation.

**Figure 4:**
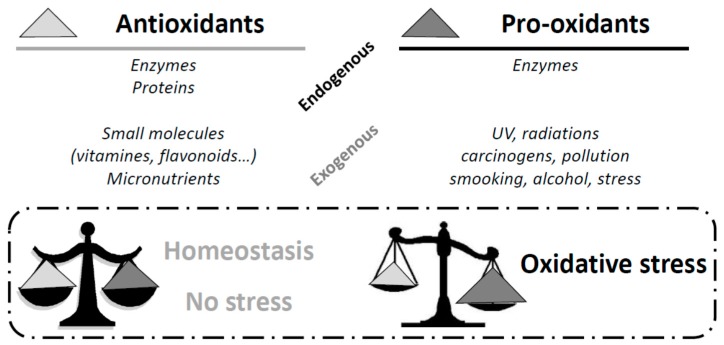
Oxidative stress: A question of balance.

Β-cells are particularly sensitive to ROS because they are low in free radical quenching (antioxidant) enzymes such as CAT, SOD, and GPx [80,81,82] and have a lower level of GSH [82,83]. However, the balance between free radicals and antioxidant defense systems is crucial to maintaining homeostasis; if its equilibrium is broken in favor of the pro-oxidant entities, pathological oxidative stress appears [84] (Figure 4).

### 3.2. Free Radicals: Good and Bad Boys?

ROS and RNS are well recognized for playing a dual role as both deleterious and beneficial species, since they can be either harmful or beneficial to living systems [85], but it is a well-known feature that cells are capable of generating endogenously and constitutively ROS [6].

#### 3.2.1. Physiological Roles: The Good Boy Side

Oxygen homeostasis at the tissue level is vital for development, growth, and survival, and cells hence have evolved a number of mechanisms to sense and respond to low oxygen levels. Under physiological conditions, beneficial effects of free radicals occur at low or moderate concentrations and involve physiological roles in the regulation of cellular signals implicated in proliferation and cell adhesion, apoptosis, inflammatory responses, and the regulation of transcription factors [6]. ROS, in low concentration, are generating when cells are stimulated by cytokines, growth factors, and hormones [86], and ROS can thus play a role as a secondary messengers [87,88] like the mitogen-activated protein kinase (MAPK) pathways [89], probably the most significant effect of metals and ROS. This involves the activation of nuclear transcription factors and control of the expression of protective genes that repair damaged DNA, power the immune system, arrest the proliferation of damaged cells, and induce apoptosis [89]. Cell adhesion plays an important role in embryogenesis, cell growth, differentiation, wound repair, and others, depending on redox regulation [90] and the involvement of NADPH oxidase [91]. In an inflammatory environment, activated neutrophils and macrophages produce a large quantity of ROS via NADPH oxidase and myeloperoxidase. This “oxidative burst” plays a key role in the defense against environmental pathogens [92]. Low and moderate levels of ROS also play important roles in regulating autophagy and apoptosis, therefore controlling cell death and survival [93,94], and ROS generated during ischemic preconditioning (alternation of short periods of ischemia and reperfusion) confer cardiac protection by reducing necrosis and the severity of arrhythmias, improving functional recovery when challenged with a longer period of ischemia [95]. This mechanism is very complex and involves triggers, mediators, and multiple second messengers’ pathways [96,97,98], but it is an innate physiologic adaptive process against potentially lethal ischemic injury. NO stimulates soluble guanylyl cyclase, leading to the relaxation of vascular smooth muscle [99] and the essential role of NO in endothelium-induced relaxation was discovered by Furchgott and Zawadzki in 1980 [100]. Nowadays, various studies report a pivotal role of NO on vascular homeostasis (anti-thrombotic, anti-aggregate, anti-migration, and relaxation) [101,102,103]. ROS play a crucial role in the activation of mechanotransduction signaling pathways and in cardiac contraction and relaxation [104]. In addition, in cardiovascular health, insulin sensitivity plays a vital role, and ROS intervene in the insulin signaling pathway. H_2_O_2_ induces typical metabolic actions of insulin, linking ROS to insulin [105], increases glucose uptake in adipocytes and muscles [106], is involved in the modulation of vascular endothelial function [107], and stimulates GLUT4 translocation and lipids synthesis in adipocytes [108]. However, ROS levels are the major determinants of impaired versus enhanced insulin sensitivity [109] through a ROS-induced increase in PI3K/Akt signaling [110].

#### 3.2.2. Pathological Roles: The Bad Boy Side

**Figure 5:**
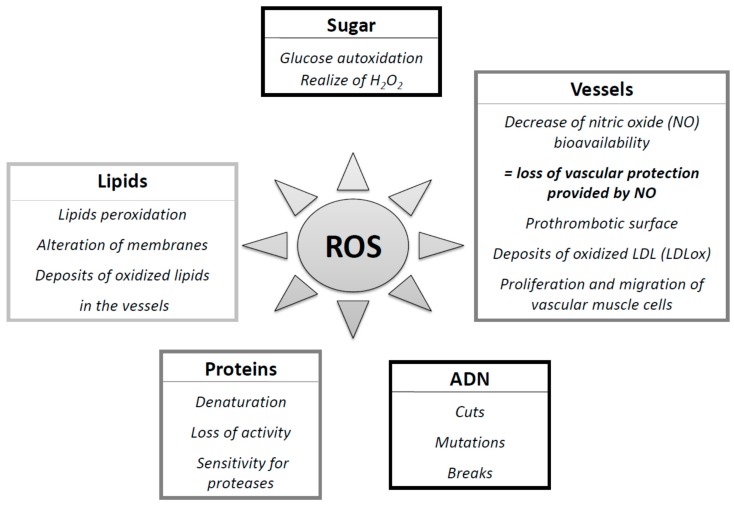
Reactive oxygen species (ROS) and complications. Impact of ROS on lipids, DNA, proteins, glucose, and vessels.

Certainly necessary in many physiological pathways, their excessive production causes direct damage to biological molecules (DNA oxidation, proteins, lipids, and carbohydrates) as well as secondary damage due to cytotoxic and mutagenic character of metabolites released in particular during the lipid oxidation (Figure 2 and Figure 5). The body may also react against these abnormal compounds by producing antibodies, which unfortunately may be autoantibodies creating a third wave of attack.

While DNA is the memory of all the biochemical live composition, it is very sensitive to free radical “attack.” At the very least, five main classes of oxidative damage mediated by OH• can be generated. Among them are oxidized bases, abasic sites, intra-catenary adducts, strand breaks, and DNA-protein bridges [111]. In addition to ROS, RNS such as peroxynitrites and nitric oxide have also been implicated in DNA damage [112]. The most extensively studied DNA lesion is the formation of 8-OH-G, and these changes are the first steps of carcinogenesis [85]; it is no coincidence that the carcinogenic agents are powerful free radical generators (UV and ionizing radiation, smoke, alcohol, asbestos fibers, carcinogenic metals, polycyclic hydrocarbons, etc.) (Figure 5).

The carbon reactive compounds (RCCs), such as malondialdehyde (MDA) and 4-hydroxynonenal (2-HNE), are formed endogenously during lipid peroxidation and glycoxidation of carbohydrates. They react with the tissue and cellular proteins to form AGEs (advanced glycation end-products) and ALEs (advanced lipid peroxidation end-products), inducing protein dysfunctions (loss of activity, increased sensitivity to proteases) [113,114] and damage in cellular responses—in particular, in inflammatory responses and apoptosis [114,115]. Lipids, mainly polyunsaturated fatty acids, are the main target of the attack by OH• and form conjugated diene radical [116]. These modifications concern circulating lipoproteins or membrane phospholipids. These derivatives are often hydrophobic and will therefore form in and around abnormal clusters of endothelial cells. These RCCs, MDA, 4-HNE, or oxidized-LDL were found in large quantities during mechanisms of carcinogenesis in various stages of cardiovascular diseases [117] such as atherosclerosis [118,119], metabolic syndrome [7], diabetes and complications [120], obesity, and insulin resistance [121], and in chronic inflammatory diseases such as lupus [122], asthma, chronic inflammation of the lungs, and respiratory allergies [123,124], and in degenerative diseases [120] (Figure 5).

### 3.3. Oxidative Stress, Diabetes, and Vascular Complications

Increased oxidative stress has been proposed to be one of the major causes of hyperglycemia-induced triggers of diabetic complications, implicates several mechanisms [125], and is a bipolar process. The first is the generation of ROS, and the second is a decrease in plasma antioxidants such as vitamin E, vitamin C, lipoic acid, and glutathione [126]. Both have been observed in diabetic patients [127,128] with micro- and macrovascular diabetic complications [3,129], linking metabolic-generated ROS to the development of diabetic complications [24]. This role of hyperglycemia has been established by large-scale prospective studies for both T1D and T2D, the DCCT/EDIC (*Diabetes Control and Complications Trial*) [130], the UKPDS (*UK Prospective Diabetes Study*) [131], and the Steno-2 study [132]. Diabetic cardiovascular complications appear to be multifactorial in origin [133,134], but, in particular, glycol-oxidative stress has been suggested to be the unifying link between the various molecular disorders in diabetes mellitus [59,135]. In fact, it is well established that hyperglycemia and acute glucose fluctuations have many side effects: modifying the redox balance, increasing circulating FFA, increasing NADPH oxidase activity and TNFα [126], and decreasing NADPH levels and glutathione, all of which generate by-products, activate oxidative, and inflammatory signaling. Hyperglycemia induces (1) an increase in glucose and other sugar fluxes through the polyol pathway, (2) an increase in advanced-glycation end-products (AGEs) formation through the hexosamine pathway, (3) expression of their receptor (RAGE) [136], and (4) the stimulation of protein kinase C (PKC) pathway. These mechanisms lead to increase production of glycative, glycoxidative, and carbonyl free radicals [22,137,138], which altered enzymatic and non-enzymatic antioxidant defenses. For example, oxidative stress increases mitochondrial DNA damages and causes axons cell death, leading to neuropathies [139]. Accumulation of sorbitol, due to an enzymatic conversion of excessive glucose, disrupts osmotic balance [140], a higher fructose production induces AGEs formation [141], and all participate in peripheral insulin resistance development [142,143] and β-cells injury [144]. Elevated AGEs may be a significant risk factor for T1D [145] and induce the progression of pre-diabetes to diabetes [146] and some complications such as diabetic retinopathy [147]. As shown before, oxidative stress is closely link to inflammation. Indeed, circulating TNF-α may impair vascular function by altering the balance between endothelial-derived vasodilator and vasoconstrictor substances because it downregulated the expression of eNOS and upregulated ET-1 production in endothelial cells [148]. Moreover, it may also directly activate NADPH oxidase and then increase the production of ROS in the vasculature [149].

Oxidative stress can be measured in vivo in multiple types including cells, solid tissues, urine, blood, and saliva. Several investigations correlated oxidative stress observed in serum and in saliva, and, today, saliva can be considerate as an oxidative stress diagnostic fluid [150,151]. Some human studies highlight reactive compounds in saliva in some pathologies, such as T1D [152,153,154,155] and T2D [153,156], with the detection of biomarkers such as 8-oxodG [152], MDA, and TBARS, proteins carbonyl [152], and total antioxidant capacity [153,154,155,156]. Recently, Wang et al. published a critical review of salivary biomarkers of oxidative stress [157], highlighting the problem of standardization in methods of saliva collection and measurements of composition. They proposed a guideline that could assist in discovery and validation of salivary oxidative stress biomarkers, allowing a diagnosis or even a simple predictive test of diabetes.

### 3.4. Endothelial Dysfunction, Diabetes, and Complications

As shown before, a large amount of evidence has demonstrated that hyperglycemia plays an important role in the pathogenesis of microvascular complications [158]. Dysfunction of the vascular endothelium is also regarded as an important factor [159,160], closely related to hyperglycemia and more recently to hypoglycemia [161], and has gained increasing attention in the study of vascular disease [162,163]. In fact, the endothelium is in constant interaction with the blood and subjected to mechanical stresses in the vessel, namely, intraluminal pressure, variations of flow including shear stress, and high glucose concentration. This strategical localization allows it a protective role as a detector toward theses stimuli. Endothelial cells respond to them through the production of messengers, addressed to cells by the blood. Thus, the endothelium plays a key role in vascular homeostasis by regulating the balance between relaxing and contracting factors. However, this protective role of the endothelium is also the first target of risk factors such as high cholesterol or high blood pressure [164], smoking [165], obesity and visceral fat distribution [166], impaired fasting glucose and hyperglycemia [167,168], insulin resistance [169,170,171,172] where this strategic balance is lost in favor to pro-mitogenic, pro-aggregation mediators [173,174], and inflammation [175]. Inflammation, in addition to oxidative stress, cause injury in cells (e.g., endothelial cells), leading to endothelial dysfunction [176] reported in numerous human and animal studies. In turn, this dysfunction promotes a pro-inflammatory environment as evidenced by increased endothelial expression of adhesion molecules, the imbalance of arachidonic acid metabolites, and chemoattractant molecules [176]. Forming a positive feedback loop, vascular inflammation leads to endothelial dysfunction (176). Lipopolysaccharide (LPS) from the bacterial cell wall [177] and C-reactive protein [178] are strong triggers for inflammation and endothelial vascular dysfunction in humans, as observed in T2D [179,180].

These disorders enable endothelial dysfunction as an early step in pathologies such as atherosclerosis and heart failure [181,182,183,184,185,186] and aging [187], as well as metabolic syndrome [188,189] and diabetes [190,191]. Endothelial dysfunction has been associated in several regions of the vasculature in animals and humans with T2D due to defects in NO-derived vasodilation [192,193], associated with diabetic complications such as nephropathy [194], retinopathy [195], and erectile function in animal models or human [190,196], and associated with cardiovascular and all-cause mortality in diabetic patients [191]. However, vascular complications may also be related to defects in endothelium-derived hyperpolarizing factor (EDHF) [193], which is thought to be an extremely important vasodilator substance, notably in resistance vasculature [64]. Unfortunately, the nature and, indeed, the very existence of EDHF remain obscure. Potentially, there are multiple EDHFs demonstrating vessel selectivity in their actions [197].

**Figure 6:**
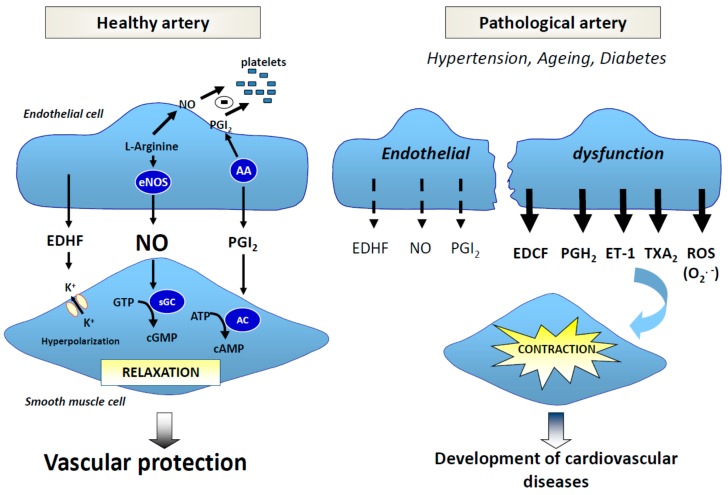
Role of endothelium in vascular homeostasis. In a healthy artery, vasodilators factors such as nitric oxide (NO), endothelium-derived hyperpolarizing factor (EDHF), and prostacyclin (PGI_2_) play a key role in homeostasis. In a pathological artery, they decrease in favor of contractor factors such as endothelium-derived contracting factor (EDCF), prostaglandin (PGH_2_), endothelin-1 (ET-1), and thromboxane A_2_ (TXA_2_) in the presence of oxidative stress and superoxide anions (O_2_^.−^). AA: arachidonic acid, eNOS: endothelial nitric oxide synthase, sGC: soluble guanylate cyclase; AC: adenylate cyclase; K^+^: potassium.

Mechanisms are complex and multiple, and etiologies are still at the heart of current research; however, oxidative stress are the common denominator [198] (Figure 6).

#### 3.4.1. Free radicals, NO and NO Synthases

Free radicals are able to modify relaxation or contraction balance in favor of contracting factor release, playing a primordial role in vascular pathologies [198]. O_2_^.−^ decreases NO bioavailability, forms peroxinitrites [199,200], and inhibits activity and expression of soluble guanylate cyclase (sGC) [201,202,203]. Peroxinitrites themselves at a high concentration inhibits sGC, prostacyclin production through the nitration of the prostacyclin synthase, inhibits SOD [202], notably in diabetes [204], and uncouples NO synthase, leading to O_2_^−^ synthesis. Peroxinitrite has a toxic effect on vasculature and contributes to the disease progression and myocardial damage [205]. This loss of NO availability induces disorders [57] such as the formation of a thrombogenic surface in the vessels, an increase in endothelium permeability and an accumulation of oxy-LDL, an attraction of monocytes and T lymphocytes, smooth muscle cell proliferation, and vascular wall growing, leading to vasculopathies. Deficiency of vascular NO is also associated with altered vasorelaxation in arterial pressure [206,207], atherosclerosis [208], hypercholesterolemia [209,210], vascular aging [62,211,212,213], metabolic syndrome [189], and diabetes [214,215]. Moreover, this blunted-NO availability is believed to be the primary defect that links insulin resistance and endothelial dysfunction [171], and is associated with oxidative stress, for example, in mesenteric arteries from established T2 models Otsuka Long–Evans Tokushima fatty (OLETF) rats [216].

In diabetes, the underlying mechanisms seem to be diverse, but include the effects of hyperglycemia [217], AGEs [211,214,218], uric acid [219], and oxidative stress [213] (Figure 6), and polymorphisms in eNOS lead to NO deficiency [220]. In fact, a high level of glucose induces an uncoupling of eNOS [221], and, although translocation to the membrane operates, this might be an inactivated form of the enzyme [222]. eNOS is not the only form to play a role in diabetes and its complications. In fact, NOS-opathies include three isoforms: neuronal (nNOS; NOS1), inducible (iNOS; NOS2), and the most well studied endothelial (eNOS; NOS3). Deletion of all three in mice results in spontaneous coronary artery diseases, myocardial infarction, and sudden cardiac death, [223,224] and results confirmed a protective role of eNOS and nNOS, whereas iNOS was found to exert an unfavorable role. Khanna et al. recently reviewed the implication of isoforms in diabetic cardiomyopathy and highlighted the important role of epigenetic modifications in the regulation of gene expression [225]. nNOS, originally expressed throughout the central and peripheral nervous system, is sympathoinhibitory in a range of diseases including chronic heart failure, chronic renal failure, and hypertension [226]. Moreover, nNOS, expressed also in macula densa cells and pylor, is involved in the pathogenesis of renal hemodynamic changes [227] and gastropyloric dysfunction [228] associated with diabetes. However, a characteristic feature of iNOS is its lack of expression in strictly resting cells. Instead, it is induced by immunological stimuli, which led to its original designation as inducible NO synthase [229]. The host cell localization of iNOS has been mainly investigated in macrophages, neutrophils, and smooth muscle cells, where the production of NO is more robust (µm vs. nM for eNOS and nNOS), continually (some days vs. min.) The authors of [230] initially intended to compensate the downregulation of eNOS by oxidative stress [231]. However, like a double-edged sword, the inflammatory cytokines, importantly, TNFα and C-reactive protein at the same time, will activate NADPH oxidase, which in turn produces O_2_^.−^. Excessive NO concentration produced reacts with O_2_^.−^ forming peroxynitrite and contributes to an uncoupled iNOS due to the substrate limitation, and therefore the production of ROS [232]. Therefore, the link of oxidative stress and inflammatory response leads to decreased NO bioavailability causing endothelial dysfunction and contractile dysfunction [233], as shown in diabetic complications [234,235,236].

#### 3.4.2. Free Radicals and EDHF

Alterations of EDHF signaling are also associated to animal and human pathologies [237], including hypercholesterolemia, arterial pressure [64], obesity [238], diabetes [239], and aging [62,213] and are characterized by O_2_^−^ induce blunted EDHF-mediated relaxations through a decrease in potassium channels sensitive to calcium (SK_Ca_ and IK_Ca_) [62,63,64,213] and myoendothelial gap junctions between endothelial cells and smooth muscle cells [64]. In mesenteric arteries from established T2D models such as OLETF-rats [240] and the insulin-resistant fatty Zucker rats (ZDF) [241,242], EDHF-mediated relaxation decreases due to alterations of both potassium channels, recently associated with oxidative stress [216,243,244] and probably involving renin-angiotensin-aldosterone systems (RAAS) [216] such as aging [62,213].

#### 3.4.3. Free Radicals and Contractions

There is great heterogeneity in the formation of EDCF (*endothelium-derived contracting factor*)-dependent stimuli, vascular beds, age, and experimental animal models used. Among contractor factors produced by endothelial cells, we cite derivatives of arachidonic acid metabolism such as endoperoxides, thromboxane A2 (TXA_2_), prostaglandin H2 (PGH_2_), and prostacyclin (PGI2), but also superoxide anions (O_2_^−^), endothelin 1 (ET-1), and angiotensin II [245]. ET-1 is increased in metabolic syndrome [189] and obesity [238], and EDCF-mediated contraction is also exacerbated by obesity, hypertension and diabetes (e.g., OLETF-rats [216]) and thus are likely to contribute to the endothelial dysfunction [246].

#### 3.4.4. Iron and Non-Transferrin-Bound Iron (NTBI)

Sometimes, the complex interactions between iron, oxidative stress, inflammation, and diabetic complications [247] have attracted considerable interest despite a poor understanding of the mechanisms involved. Numerous forms of body iron exist, and only forms not bound to transferrin or other iron-binding proteins named non-transferrin-bound iron (NTBI) seem to be implicated in oxidative damages due to their high reactivity [248]. NBTI could be considered a biomarker of the side effect of iron in diseases, greatly correlated with Hb1Ac [249]. Recently, Aljwaid et al. [249] confirmed association of NTBI with the risk of vascular complications in diabetes already highlighted 10 years earlier [250,251,252], because NBTI is easily accessible to plaque as well as endothelial cells, macrophages, and smooth muscle cells. Inflammation contributes to iron-mediated endothelial dysfunction, characterized by a high release of iron by infiltrated macrophages, an increase in E-selectin, and other adhesion molecules implicated in atherosclerotic plaque [247,253]. Iron can enter into the atherosclerotic lesion in the form of free hemoglobin, which is prone to oxidation, and can form methemoglobin, ferryhemoglobin, and release heme. All of these exert pro-oxidant and pro-inflammatory effects on the vascular wall [253]. Vinchi et al. [253] summarized current knowledge about the role of hemoglobin, heme, and iron through controversial epidemiological studies and concluded, given more evidence, their negative impact, compared with the innocent role of iron in atherosclerosis. The chronic increase in the release of hemoglobin and heme (hemolysis) is associated with endothelial dysfunction and reduced NO bioavailability [254] and with coagulopathy [255,256] and vasculopathy [256], as observed in diabetes [257], greatly reviewed by Vinchi et al. [258].

## 4. Nutritional Prevention: Antioxidants against Diabesity and Complications

Regarding the low level of antioxidant enzymes expression in the pancreas [80], combinations of conventional antidiabetic treatments with antioxidants were quickly privileged [259]. A Mediterranean diet (MedD) is characterized by abundant plant foods (fresh fruit, vegetables, breads, other forms of cereals, seeds, etc.), olive oil as the principal source of fat, and wine. The PREDIMED study examined the effect of a one-year MedD on oxidative and inflammatory parameters in subjects with a high risk for cardiovascular diseases. Results showing that the MedD increases plasma non-enzymatic antioxidant capacity, decrease the biomarkers of atherosclerosis,have anti-inflammatory effect in addition to the improvement of lipid profile, insulin sensitivity, blood pressure, and carotid atherosclerosis. Adherence to MedD reduces the incidence of T2D, metabolic syndrome, and diabetic retinopathy. However, the MedD have no effect on diabetic neuropathy, highlighting complexity to recommend an ideal model for diabetic complication prevention. In patients with newly diagnosed T2D, consumption of this diet resulted in a greater reduction of HbA1c levels, a higher rate of diabetes remission, and delayed need for diabetes medication [260]. Moreover, a Mediterranean diet enriched with extra-virgin olive oil but without energy restrictions reduced diabetes risk among persons with a high cardiovascular risk [261]. Antioxidants act synergistically or by trapping single electrons to free radicals or by reducing ROS enzymatically. Some antioxidants such as vitamins E (tocopherol), C (ascorbate), and Q (ubiquinone), and carotenoids or polyphenols come from food. Inhibition of hyperglycemia-induced ROS production using transgenic antioxidant enzyme expression or antioxidant compounds prevents the development of experimental diabetic retinopathy [262], nephropathy [263,264], neuropathy [265], and cardiomyopathy [266]. Additionally, the mechanisms behind the anti-inflammatory effect of carotenoids (β-carotene and lycopene) have been recently described: both decrease TNFα-mediated ROS generation and increase NO bioavailability at the endothelial level, linking oxidative stress inflammation and vascular beneficial impact [267]. In humans, some large epidemiological studies such as the Linxian study, the Clark study, the Qixia study, the NPC study, or the SU.VI.MAX study in France, the feasibility and efficacy to prevent cancer or mortality with moderate doses of antioxidants has been demonstrated in healthy subjects. Zatalia et al. [16] recently listed all the beneficial effects observed in animals and humans, from vitamins and supplements, plants but also drugs used for treating diabetes and its complications. These experimental and human studies led to a proposal for nutritional prevention to inhibit diabetic complications. Table 1 resumes some classical products that have potential cardiovascular protective effects.

We will now see different management strategies of diabetes and complications using non-exhaustive examples of the interest inspired by plants, fruits and vegetables, polyphenolic compounds, and even some drugs used today in the treatment of diabetes with an antioxidant activity (Table 2, Table 3, Table 4, Table 5, Table 6 and Table 7).

### 4.1. Plant Therapy

Plants have been used from a long time by Chinese, African, and South American peoples as traditional medicines and is used by about 60% of the world’s population. The first texts written about herbal medicine are etched in clay. It includes a series of tablets engraved in cuneiform, and its authors, the Sumerians, drafted them 3000 years before the common era. They used plants such as myrtle, hemp, thyme, and willow. From century to century, Theophrastus, Aristotle, Pliny the Elder, and Dioscorides deepened their knowledge of plants and their properties. Morphine, aspirin, quinine: What do they have in common? All come from nature and have led to major drugs. Morphine is extracted from opium (*Papaver somniferum*), aspirin is extracted from willow bark, and quinine is from a tree from the Cordilleras in the Andes called the cinchona. The world contains many molecules with interesting biological properties, but they must be highlighted. Recently, there has been considerable interest in finding natural antioxidants from plant materials to replace synthetic ones, and natural antioxidants occur in all higher plants and in all parts of the plant (wood, bark, stems, pods, leaves, fruit, roots, flowers, pollen, and seeds) [269]. There have been many investigations into the effects of these plants and their antioxidant ingredients on diabetes and its complications, and good results have been achieved. Dixit et al. focuses on Indian Herbal drugs and plants used in the treatment of diabetes, especially in India [270]. Dodda and Ciddi [15] reported on other plants used in the management of diabetic complications (nephropathy, neuropathy, cataract, and retinopathy) and, last year, Qiang et al. [271] demonstrated the protective effect of *Sancaijiangtang* on NO and ET-1 dysfunction observed in the vessels of T2D patients. Table 2 shows antioxidant properties of some of these plants, except from those treated by Dixit in his review.

If herbal medicine enjoys an extraordinary craze across the world, this is not just a matter of fashion. Of course, our era is deeply marked by the search for a healthier life, a return to nature and essential values. One recent example is the use of *Stevia*, with 200 species around the world growing primarily in the Amambay mountain range of Paraguay [272]. S*tevia rebaudiana,* the only species with the ability to sweeten with no caloric value, contain specific substances (glycosides) in leaves that are rich in vitamins and complements [273]. Research on diabetic rats has shown the antihyperglycemic, insulinotropic, and glucagonostatic actions of *stevia* [274] and its ability to reduce postprandial blood glucose levels in type 2 diabetic patients, indicating its beneficial effects on glucose metabolism [275]. *Stevia* offers an ideal alternative to sugar, well tolerated, with a zero glycemic index and no pharmacological effect in T1D and T2D patients [276].

### 4.2. Fruits and Vegetables

Scientific and medical interest in cardiovascular health benefits of fruit- and vegetable-rich diets has grown exponentially in recent years, due to compelling epidemiological evidence showing that the consumption of fruits and vegetables might reduce the risk of cardiovascular diseases [10,11,12,13,14]. Although studies demonstrate no significant beneficial effect against diabetes [293], others highlight a decrease in the risk to develop diabetes [294,295], which was confirmed by a recent meta-analysis on diets rich in green leafy vegetables [296]. Their antioxidant capacities in humans have also been demonstrated in many studies, namely, the effects of strawberries and tomato juice on metabolic syndrome, hyperlipidemia, and T2D [297,298,299]. Table 3 shows experimental studies that evaluate the effect of natural antioxidant products, fruits, and vegetables on diabetes and its related complications.

Recently, studies suggested that these beneficial effects could be due to nitrate content [318,319,320]. Machha and Schechter [321,322] reviewed and reported the beneficial effects of nitrite and nitrate on cardiovascular health, especially with respect to vascular function. Nitrites and nitrates, the content of the fruits and vegetables [323] and direct eNOS subtracts, can improve NO bioavailability in the vasculature and improve endothelial function and all the beneficial effects of NO, nitrites and nitrates as a substrate to eNOS. This evidence has been shown by several in vitro and in vivo animal models [324,325,326] and in humans [325,327,328] to increase the bioavailability of NO to reduce vascular tone, blood pressure, and micro- and macrovascular complications, and improving insulin sensitivity is certainly an attractive therapeutic target in T2D.

Even though antioxidant and anti-inflammatory mechanisms by which fruits and vegetables exert their protective effects are not entirely clear, some studies have identified several bioactive components such as carotenoids, vitamins, fiber, magnesium, and potassium as acting synergistically or antagonistically to promote a holistic beneficial effect. For example, vitamin C restores endothelial function in T1D patients, leading to decreased micro- and macrovascular complications [329]. Chronic vitamin E, with low (100 UI/d, 3 months) or high (250UI/d, 6 months) doses, decrease lipid peroxidation in T1D patients [330,331]. Vitamin E is the best example that shows the complexity of antioxidant studies. In fact, this antioxidant supplement has been investigated extensively. Since 1998, Heinonen et al. [332] has suspected an increase in prostate cancer, not confirmed later by Lippma et al. [333] and Gaziano et al. [334] in 2009. However, in 2005, Miller et al. [335] described an increase in all-cause mortality, and the SELECT study was stopped in 2008 due to an increase in prostate cancer with 400UI/d of vitamin E [336]. Moreover, a randomized clinical trial with vitamin E showed no cardiovascular benefits, mainly in non-diabetic subjects [337]; this was confirmed later by a HOPE clinical trial [338,339]. However, an analysis of all data in a sub-group of subjects with diabetes and haptoglobin 2-2 genotype in HOPE and ICARE studies revealed that, in fact, vitamin E (400UI/d, 18 months) reduced the rate of cardiovascular events in these high risk subjects [340,341], which was confirmed in a recent meta-analysis [342].

Table 4 shows antioxidant efficacy of vitamins and supplements focus in diabetes and its complications.

### 4.3. Polyphenols: Extract Versus Molecular Compound

Polyphenols are a large and heterogeneous group of phytochemicals of plant-based foods, including tea, coffee, wine, cereal grains, vegetables, legumes, fruits, and berries [372]. And the largest and best-studied polyphenols are flavonoids, which include several thousand compounds, among them flavonols, flavones, flavonones, flavan-3-ols, anthocyanins, and isoflavones [373]. The estimated intake of dietary polyphenols is approximately 1 g/day [374]. Increasingly, the dietary recommendations for individuals at risk of T2D emphasis the intake of plant food products, such as whole grains, berries, fruits, and vegetables, all known to be excellent sources of dietary fiber, but also good sources of variable polyphenolic compounds. In fact, epidemiological studies report an inverse association between dietary polyphenol consumption and both diabetes [17,18,19,20] and more generally in chronic diseases such as cardiovascular diseases, atherosclerosis, hypertension, and cancer [375].

As shown before, vascular protection may also be due to the direct action of polyphenols on the endothelial function. In fact, polyphenols are able to stimulate the endothelial formation of NO and EDHF in isolated blood vessels, and improve endothelial function in humans. Schini-Kerth et al. [21] described the vascular protection led by natural product-derived polyphenols in ex vivo and experimental models of cardiovascular disease, including metabolic syndrome and diabetes. Recently, Franzini et al. [376] indicated that diets that contain a high level of polyphenol-rich natural sources such as red wine, grapefruit, berries, and dark chocolate, improved endothelial function in a low cardiovascular risk population, and Khan et al. [377] discusses the effects of cocoa polyphenols on cardiovascular-related inflammation. Table 5 shows the effect of polyphenol-rich natural sources on human vascular function.

Besides their beneficial effects on endothelial function and vascular homeostasis, they also influence glucose metabolism by several mechanisms, such as the inhibition of carbohydrate digestion and glucose absorption in the intestine, the stimulation of insulin secretion from the pancreatic β-cells, the modulation of glucose release from liver, the activation of insulin receptors and glucose uptake in the insulin-sensitive tissues, and the modulation of hepatic glucose output [410]. Many polyphenols have been shown to inhibit mostly α-glucosidase activity in vitro (anthocyanins, catechins, flavanones, flavones, flavanols, isoflavones, phenolic acids, and proanthocyanidins), whereas α-amylase activity is inhibited only by phenolic acids and some flavonols such as quercetin, luteolin, and myricetin. As regards the various effects of polyphenols, very few of them are able to induce insulin secretion from cultured cells or islets isolated from pancreas (cyanidin and delphinidin, epicatechin and EGCG, rutin, quercetin, apigenin, etc.) and inhibit the sodium-dependent glucose transporter (SGLT1) and the glucose transporter GLUT2) (tea catechins and quercetins) [410]. Recently, Hanhineva et al. [410] listed the impacts of dietary polyphenols on glucose metabolism with in vitro and in vivo studies and highlight the protective role of dietary rich in polyphenols on carbohydrate metabolism in both animals and humans. For example, Rostami et al. [411] demonstrated that cocoa is effective in improving TG levels, decreasing blood pressure, and fasting blood sugar in T2D patients with hypertensive complications. A meta-analysis of eleven randomized controlled clinical trials showed that resveratrol significantly improves glucoregulation and insulin sensitivity in diabetic patients, but not control participants [412]. Similar results were obtained in a second meta-analysis that included only T2D patients [413]. One recent review [414] reported the latest advances regarding the timing, dosage, formulation, bioavailability, toxicity of resveratrol in human, focusing on cancer, neurogeneration and diabetes, obesity, and cardiovascular diseases. Curcumin has been reported as a potent scavenger of a variety of ROS [415], exhibiting anti-inflammatory activity as well as antioxidant properties [416]. The phenolic (OH) structure of curcumin was believed to be essential for curcumin’s anti-oxidant activity [417]. Novelle et al. [414] concluded about difficulties of establishing a specific range of safety/efficacy for particular doses of resveratrol for particular populations, and many discrepancies and conflicting information must be resolved before recommending the use of resveratrol. Table 6 and Table 7 show the effect of polyphenol-rich natural sources on human prevention of T2D on in vitro and in vivo models of diabetes and complications, respectively.

### 4.4. Current Medications

Some modern drugs are derived from traditional medicine: anti-malarials (artemisinin, quinine), anti-asthmatics (cromolyn), anti-cancer (etoposide, vinca alkaloids), anti-coagulants (huridine), anticholestérolémiants (Lavastatine), and analgesics (opiates) [454]. Moreover, drugs used to treat diabetes have an antioxidant activity [16]: a scavenger of ROS and a modulator of antioxidant enzymes activities by several mechanisms. Some of them have beneficial effects on diabetes complications such as nephropathy—angiotensin converting enzyme inhibitor (ACEI), angiotensin receptor blocker (ARB), and melatonin, and neuropathy and retinopathy—melatonin and α-lipoic acid. Many of them have beneficial effects against cardiovascular diseases: caffeic acid, phenethyl ester, carvedilol, and metformin [16]. In fact, metformin, the currently used biguanide antihyperglycemic agent, can decrease xanthine oxidase activity and TNFα production, chelates metal ions, and inhibits AGE formation [455] with an intracellular modulation of free radical production [72].

## 5. Discussion and General Conclusion

In the last few years, there has been an exponential growth in the field of herbal medicine, and these drugs are gaining popularity. Many traditional medicines in use are derived from medicinal plants, minerals, and organic matter, and many conventional drugs have been derived from prototypic molecules. The use of medicinal plants for therapeutic purposes is a practice as old as human history. Some studies and population observations highlight a real effect of plants on health and management of diabetes complications. Today, the WHO has listed 21,000 plants, which are used for medicinal purposes around the world, but the expert committee on diabetes has recommended that traditional medicinal herbs be further investigated.

Nutrition and diet quality are key elements in the acquisition, control, and potential treatment of many chronic diseases and adverse health conditions. Higher consumption of fruits and vegetables has been associated with a lower risk of several diseases, including cardiovascular disease [11,12]. Increased physical activity and dietary management implemented by health-care professionals is fundamental to initial treatment of T2D and has been recommended for a long time by international consensus [456]. Meta-analyses of exercise and diet studies have concluded that concentrations of HbA1c can be lowered by aerobic and resistance exercise and by dietary intervention [457,458], more precisely, intensified, targeted, multifactorial interventions compared to conventional intervention [459]. However, few studies have determined whether treatments affect endothelial dysfunction and oxidative stress. If multifactorial treatment does have an effect, then markers of endothelial dysfunction and oxidative stress would be expected to be less associated with cardiovascular death and all-cause mortality in these patients. In fact, most of the studies have reported the beneficial effects of natural products-rich in antioxidant activities, leading to protect vessels against oxidative stress, loss of vascular homeostasis, and diabetic complications. Recent data have supported that the hyperglycemic environment may be remembered in the vasculature, a metabolic or “hyperglycemic memory” explaining the progression of diabetic vascular complications despite normoglycemia restoration [460]. Moreover, endothelial progenitor cells as a biological marker of peripheral artery disease [390] have highlighted a real interest in protecting the vascular arch [461]. Thus, taking all of this literature together, blood vessels could be a good marker and strategy to monitor complications, especially in diabetes.

Observational cohort studies support that consumption of sugar-sweetened beverages, including artificially beverages and fruit juice, are associated with incident T2D, independently of obesity. Both were unlikely to be healthy alternatives to sugar-sweetened beverages for the prevention of type 2 diabetes, and, under assumption of causality, there consumptions may be related to a substantial number of cases of new onset diabetes [462]. The local food environment may influence individual (including food choices) and community health [463]. Today, the objective is to promote the consumption of non-industrial and natural products instead of concentrated fruit juice intake. In fact, the association between fruits and vegetables consumption and weight development has been summarized in the ISA-FRUIT Project of the EU from 2008, and 7/16 studies [464] and several prospective cohort studies [296,465] and the *EPIC-Norfolk Study* [466] have highlighted an inverse association between “unworked” fruits and vegetables consumption and health outcomes including obesity, cardiovascular, and diabetes. However, others studies have not demonstrated the effectiveness of fruits and vegetables to have health effects or to prevent chronic diseases. These results suggest that there are sub-types within larger categories of food environments that are differentially associated with adverse health outcomes [467]. Differences in the nutrient contents by group could explain differences and raise difficulties of interpreting the results of different human studies. There is a need to conduct clinical research, developing simple bioassays for biological standardization, pharmacological, and toxicological evaluation, to study the effects of natural food on health.

It would actually be profitable to propose tables’ effects of antioxidants, with corresponding doses and diseases treated; still, however, the study of antioxidants is very difficult and complex. Many parameters can influence the results of clinical studies: a different design in terms of types and origins of antioxidants, doses, formulation, absorption, biodisponibility, and times of treatments; the studied population, genotype sub-type of patients, types of medication, and progression of the disease, with a time course of diabetes and complications; and the methods of assessment and their limitations [468,469,470]. The international society of antioxidants in nutrition and health (ISANH) work today to propose guidelines with all these objectives. Although the results of clinical studies about the therapeutic use of antioxidants are quite controversial, all data reported in this review and in others provide real hope for their use, especially in the prevention of diabetic complications. Food is the first pillar of patient care before the introduction of medications. Wealth, nutritious additions, and a contribution of bioactive molecules (vitamins, polyphenols, etc.) with antioxidant properties is actually a real asset in the prevention of chronic diseases, while the importance of prevention should not be underestimated. All publications by Dal et al. demonstrated the interest of the use of natural antioxidants (red wine polyphenols) to prevent and treat diseases with endothelial dysfunction related to oxidative stress [62,64,213,471,472]. Moreover, our recent works carried out on in vitro and in vivo models of metabolic disorders have allowed us not only to involve oxidative stress in the pathophysiology of disorders but also to demonstrate that natural antioxidant compounds help to prevent or reduce complications (polyphenols from green tea and red wine [442,473], red cabbage, Dal S, under publication). One study in process in the lab focusing on antioxidants in T1D seems be a new target for the diabetic optimization of management [474].

All of our studies, and studies mentioned in this review, demonstrate the ability of antioxidants to prevent or counteract excessive ROS production by increasing endogenous antioxidant defenses. We think now that a new strategy might be to prevent the overproduction of ROS instead of only scavenging the already formed ones, because of the “cardio-metabolic memory.” Today, an optimal understanding of the beneficial mechanisms of functional products or functional foods [475] will not allow for more personalized care, depending on the status of cardiovascular and metabolic patients.

## Figures and Tables

**Table 1:** Effects of functional foods and their bioactive compound on cardiovascular parameters [268].

Functional Foods	Bioactive Compound	Mechanisms
Black tea	Tea polyphenols Anthocyanins, catechins, cyanidins	↓ blood pressure
Citrus fruit	Vitamin C Ascorbic acid	Inhibition of ox-LDL ↓ blood pressure Antioxidant action Endothelial function
Dark chocolate	Flavonoïd	Lowering blood Chol Inhibition of ox-LDL ↓ blood pressure Endothelial function
Extravirgin olive oil	Polyphenolics and oleic acid Tocopherols, tocotrienols	Inhibition of ox-LDL Antioxidant action
Fish	Omega-3 fatty acids	Lowering blood Chol Inhibition of ox-LDL Lowering blood TG ↓ blood pressure Endothelial function
Fruits and vegetables	Fibers (pectin) Carotenoids Vitamin C	Lowering blood Chol Inhibition of ox-LDL Antioxidant action Endothelial function
Ginseng	Ginsenosides	↓ blood pressure
Grapes and red wine	Grape polyphenols Anthocyanins, catechins, cyanidins and flavonols Myricetin and quercetin	↓ blood pressure Antioxidant action Endothelial function Platelets aggregation
Green leafy vegetables	Carotenoids	Inhibition of ox-LDL Antioxidant action
Green tea	Tea polyphenols	Inhibition of ox-LDL ↓ blood pressure Antioxidant action
Margarine	Phytosterols	Lowering blood Chol
Nuts	Tocopherols, omega-3 fatty acids Polyphenols	Lowering blood Chol Endothelial function
Onion and garlic	Quercetin	↓ blood pressure
Pomegranate	Polyphenols	Inhibition of ox-LDL
Soy proteins	Genistein and daidzein glycitein	Lowering blood Chol Inhibition of ox-LDL Antioxidant action
Tomato	Lycopene	Inhibition of ox-LDL Antioxidant action
Vegetable oil	Tocopherols, tocotrienols	Antioxidant action
Whole grains	Fibers and phytochemicals	Lowering blood Chol ↓ blood pressure

Chol: cholesterol; ox-LDL: oxidation of LDL; TG: triglycerids.

**Table 2:** Effects of plants on oxidative and metabolic parameters.

Plants	Experimental studies	Efficacy
*Allium cepa* *Allium sativum*	Alloxan-induced diabetic rats [277] and STZ-induced diabetic rats [277]	• ROS scavenger • ROS scavenger • ↓ oxidative stress (lipid peroxidation) • ↑ SOD, ↑ GST
*Aralia elata*	STZ-induced diabetic rats [277]	• Inhibition of aldose reductase • Inhibition of cataract (retinopathy)
*Aloe verra*	STZ-induced diabetic rats [277]	• ↑ antioxidant enzymes activities • ↓ oxidative stress (lipid peroxidation)
*Anoectochilus formosanus*	STZ-induced diabetic rats [277]	• ↑ antioxidant enzymes activities • ↓ oxidative stress (lipid peroxidation)
*Cassia fistula*	Alloxan-induced diabetic rats [277]	• ↑ antioxidant enzymes activities • ↓ oxidative stress (lipid peroxidation)
*Coccinia indica*	STZ-induced diabetic rats [278,279,280]	• hypoglycaemic/hypolipidaemic effects • ↑Vitamin C, antioxidant activity • ↑ antioxidant enzymes activities
*Eugenia jambolana*	STZ-induced diabetic rats [277]	• ROS scavenger
Ever green shrubs (*Larrea divarita*)	STZ-induced diabetic rats [277]	• ↓ XO activity, ion chelation, ROS scavenger, ↓ blood pressure, inhibition of nephropathy
*Fomes fomentarius*	STZ-induced diabetic rats [277]	• ↑ antioxidant enzymes activities • ↓ oxidative stress biomarkers
*Juglans regia*	T2D-mouse [277]	• ↓ oxidative stress biomarkers
*Trigonella foenum-graecum (fenugreek)*	T2D patients [281]	• Hypoglycemia effect
*Lycium barbarum*	Alloxan-induced diabetic rats [277]	• ↓ lipids
*Panax ginseng*	T2D rats [277]	• ROS scavenger • Erectile dysfunction protection
*Potentilla chinesis*	STZ-induced diabetic rats [282]	• ↑ antioxidant enzymes activities • ↓ oxidative stress (lipid peroxidation) • ↓ blood glucose • ↓ LDL, ↓TG, ↑HDL
*Scoparia dulcis*	STZ-induced diabetic rats [277]	• ↑ antioxidant enzymes activities • ↓ oxidative stress biomarkers • ↑ GSH
*Stevia rebaudiana bertoni*	STZ-induced diabetic rats [283]	• ↓ blood glucose, ↑glucose tolerance • ↑ insulin levels and ↑sensitivity • ↓ALT, ↓AST, ↑ filtration rate glomerular • Improve kidney damages (nephropathy) • ↓ oxidative stress (lipid peroxidation) • ↑ total antioxidant capacity • ↑ antioxidant enzymes activities
*Trifolium alexandrium*	STZ-induced diabetic rats [277]	• ↑ antioxidant enzymes activities • ↓ oxidative stress (lipid peroxidation)
*Ulva lactuca polysaccharides (alga)*	STZ-induced diabetic rats [284]	• ↓ blood glucose • ↓enzymes of lipid metabolism and absorption • ↓ LDL, ↓TG, ↑HDL • protection hepatic and renal functions
*Vitis vinifera*	Alloxan-induced diabetic rats [277]	• ROS scavenger • ↑ GSH • ↓ oxidative stress (lipid peroxidation)
*Viburnim dilatatum*	STZ-induced diabetic rats [277]	• ROS scavenger • ↓ oxidative stress (lipid peroxidation)
*Viscum album*	STZ-induced diabetic rats [277]	• ↑ antioxidant enzymes activities • ↓ oxidative stress biomarkers
Nopal (*Opuntia streptacantha Lemaire*)	Healthy people [285] T2D patients [286,287]	• Hypoglycemia effect • ↓ blood glucose, ↓ insulin • ↑ insulin sensitivity
*Pycnogenol®*	Healthy people [288] Hypertensive patients [289] Metabolic syndrome patients [289]	• ↑ NO-mediated forearm blood flow • ↓ blood pressure • Improve endothelial function
*Zygophyllum album*	Alloxan-induced diabetic rats [290]	• ↓ blood glucose, ↓obesity
Many plants	STZ-induced diabetic rats [291]	• ion chelation, ROS scavenger • ↓ oxidative stress (lipid peroxidation)
Plants like *ferula assa-foetida*	STZ-induced diabetic rats [277] KK-Ay mice [292]	• ↑ antioxidant enzymes activities • ↓ oxidative stress biomarkers • ↓ blood glucose

ALT and AST: hepatic transaminases; GSH: gluthatione; GST: glutathione S-transferase; ROS: reactive oxygen species; SOD: superoxide dismutase; STZ: streptozotocin; TG: triglycerides; XO: xanthine oxidase.

**Table 3:** Effects of fruits and vegetables on experimental diabetes models.

Fruits or Vegetables	Experimental Studies	Efficacy
Apple	STZ-induced diabetic rats [300]	↓ TG, serum LDL and VLDL ↓ food intake ↓ weight ↓ glycemia
Asparagus	STZ-induced diabetic rats [301]	↑ pancreatic β cells functionality ↓ hyperglycemia Improves oxidative status
Black radish	STZ-induced diabetic ratsHigh Fat Diet rats [302]	↓ cholesterol and triglycerides **no effect on glycemia** ↓ oxidative stress (lipid peroxidation) Improves plasmatic antioxidative status
Celery-root	Alloxane-induced diabetic mouse [303]	↑ insulin secretion ↓ oxidative stress (lipid peroxidation) ↑ antioxidative enzymes activity (CAT, SOD, GSH)
Cherry	Alloxane-induced diabetic rats [266]	↓ glycemia Improves renal function
Cucumber	Alloxane-induced diabetic mouse [304]	↓ glycemia ↓ cholesterol and triglycerides
Garlic	STZ-induced diabetic rats [305,306]	↓ serum glycemia ↓ serum triglycerides ↓ serum cholesterol Improves endothelial dysfunction
	Alloxane-induced diabetic rats [307]	Antioxidative properties ↓ hyperglycemia
	High Fat Diet rats [308]	↓ oxidative stress (lipid peroxidation)
	Resistant rats [280]	↓ glycemia
Green bean	STZ-induced diabetic rats [309]	↓ AGEs development (↓ branched collagen)
Onion	STZ-induced diabetic rats [310,311,312]	Improves glycemia regulation Improve glucose tolerance ↓ hyperglycemia ↓ oxidative stress (TBARS, 8-OHdG)
	High Fat High Sucrose rats [313]	↓ oxidative stress (lipid peroxidation) ↓ NADH oxidase activity
Red cabbage	STZ-induced diabetic rats [314]	↓glycemia Improves renal function ↓ lipid peroxidation ↑ antioxidative enzymes activity (CAT, GPx, SOD) Improve nephropathy
Shallot	Fructose-induced Insulin resistant rats [315]	↓ glycemia
Strawberry	High Fat Diet mouse [316]	↓ inflammation (C protein CRP reactive) ↓ glycemia
Tomato	STZ-induced diabetic rats [317]	↓ lipid peroxidation ↓ glycemia Improves insulin secretion ↑ antioxidative enzymes activity (CAT, SOD, GPx)
Zucchini	Alloxane-induced diabetic mouse [304]	↓ glycemia ↑ insulin levels ↓cholesterol and triglycerides

AGEs: advanced glycation end-products; CAT: catalase; SOD: superoxide dismutase; GPx: gluthatione peroxidase; TG: tryglicerides; TBARS: peroxided-lipids.

**Table 4:** Effect of vitamins and supplements in diabetes and complications.

Vitamins	Human or Experimental Studies	Efficacy
Vitamin C	T2D patients [343,344]	↓fasting plasma insulin level, ↓HbA1c ↑insulin sensitivity
	T1D patients [329]	Restore endothelial function
	Healthy patients [343,345]	↑ insulin sensitivity ↑ endothelial function
	Diabetic rats [346]	Improve retinopathy
Vitamin D	Young predisposed child to T1D [347,348]	↓ risk for T1D
	T2D-rats [349]	↓ vascular lesions, ↓ inflammation ↓ leucocytes adhesion
Vitamin E	Diabetic patients [350]	↓ OS biomarkers, ↓insulin resistance
	T2D patients [351]	↓ OS biomarkers, ↓ ox-LDL
	T2D patients [352,353]	↓ protein glycation, ↓ROS
	T2D patients [354]	↓ ROS, ↓ retinopathy
	T2D patients [355,356,357,358]	↑ Insulin secretion, ↓ glycemia, ↓ HbA1c ↓ TG, ↓ FFA, ↓ T-Chol
	T2D patients [299,359,360]	↓ inflammation, ↑ antioxidant defenses, ↓ ox-LDL
	T2D patients [341,360]	↓ CV complication, ↑ endothelial function
	Diabetic patients [340,361]	Prevention of myocardium infarction, stroke, CV death
	T1D patients [330]	↓ lipids peroxidation
	T1D patients [362]	↓ retinal homodynamic abnormalities (retinopathy)
	Diabetic Balb/c mice [363] Diabetic rats [346]	Improve atherosclerosis Improve retinopathy
Combined with nicotinamide	IMDIAB IX study T1D children [331,364]	↑ C peptid levels
Transitional metal chelating agent	STZ-induced diabetic rats [365,366]	↓ early neuropathy ↓ hyperglycemia-induced endothelial dysfunction
Selenium	Alloxane-induced diabetic rats [367]	↑GSH in liver and brain
Zinc	STZ-induced diabetic rats [368]	↓ retinal lipid peroxidation
Combined vitamin C, E, selenium, Zinc and Β-carotene	SU.VI.MAX Healthy patients [369]	No effect on fasting glycemia ↓ cancers and death in man
B-carotene	Alloxane-induced diabetic rats [370] and T2D patients [371]	↓ ox-LDL

CV: cardiovascular; FFA: free fatty acid; GSH: glutathione; ox-LDL: oxidized-LDL; OS: oxidative stress; ROS: reactive oxygen species; STZ: streptozotocin; T-Chol: total cholesterol; TG: triglycerides.

**Table 5:** Beneficial effects of several polyphenol-rich natural sources on vessels in humans.

Natural Sources	Human Studies	Efficacy
***Plants***		
Soybean	Woman with CV risk factor [378]	↑ FMD
***Grape-derived products***		
Red wine + olive oil	Healthy people [379,380,381] Healthy people [382]	↑ basal FMD ↑ basal FMD
Red wine	Atherogenic potential [383,384] Healthy people [385]	↑ FMD, ↓ blood pressure
	Hypercholesterolemic patients [386]	improved FMD, enhanced endothelium-independent vasodilation
	Coronary artery disease [387,388]	↑ FMD
Grape juice	Healthy people [389]	↑ basal FMD
	Hypercholesterolemic patients [386]	↑ FMD protect against coronary artery disease
Concord grape juice	Coronary artery disease [390]	↑ FMD
Grape seed extract	Healthy people [391,392] Coronary artery disease [393,394] Hypertensive patients [395]	↑ basal FMD ↑ FMD ↓ blood pressure
Dark chocolate	Atherogenic potential [396]	↑ basal FMD, ↓ blood pressure
	Hypertensive patients [397,398]	↓ blood pressure
	Overweight adults [399]	↑ FMD, ↓ blood pressure (sugar-free preparations)
	Healthy people [400]	↓ blood pressure
Cocoa	patients [401] Overweight adults [399]	↑ basal FMD by 30% reverse vascular dysfunction **no effect on glycaemia control** ↑ FMD, ↓ blood pressure(may attenuate by sugar)
	Hypertensive patients [402]	**no effect on blood pressure**
Pomegranate juice	Severe carotid artery stenosis [403] Hypertensive patients [404]	↓ blood pressure, ↓artery thickness ↓ blood pressure
Strawberry	Obese patients [405]	↓ risk factors for CVD and stroke
***Teas***		
Black tea	Coronary artery disease [406]	↑ FMD
EGCG extract (Teavigo®) Green tea	Coronary artery disease [407]	↑ FMD
	Borderline diabetes or diabetes [408]	↓ blood pressure
	Healthy prospective cohort [309]	↓ CV mortality strongly vs. all cause↓ stroke
	Coronarien patients [407]	Endothelial cells protection (↑ NO) ↑ FMD
*Maritime* Pycnogenol®	Healthy people [288]	↑ NO-mediated forearm blood flow
	Hypertensive patients [289]	↓ blood pressure
	Metabolic syndrome patients [289]	Improve endothelial function
***Oil***		
Krill oil (Ѡ3 and fatty acid)	T2D patients [409]	Improve endothelial function ↑ HDL

CV: cardiovascular; FMD: flow-mediated dilatation (technic to measure endothelial function in humans).

**Table 6:** Beneficial effects of several polyphenol-rich natural sources on Human cardio-metabolic diseases.

Polyphenols	Human study	Efficacy
***Single compounds***		
Quercetin Myricetin	different national public health registers [418]	↓ risk T2D an chronic disease
Quercetin Kaemferol Myricetin Apigenin Luteolin	The Woman’s Health Study [419]	no effect
EGCG extract	Overweight or obese men [420]	**no effect on insulin sensitivity,** **no effect on glucose tolerance,** modest ↓ in DBP
	T2D patients [421]	**no effect on insulin sensitivity,**
	T2D patients [408,422]	**no effect on HbA1c and glycaemia and Insulin resistance**
Lipoic acid	T2D patients [423]	↑ insulin sensitivity
Ѡ-3	DAISY (*Diabetes Autoimmunity Study in the Young*) = predisposed T1D-children [424]	↓ risk of autoimmunity against islets, antioxidant effect
Pycnogenol®	Diabetes patients [289]	↓ blood glucose
	Hypertensive patients [289]	↓ blood pressure
	Metabolic syndrome patients [289]	↓ waist circumference, improve lipid profile, renal and endothelial functions
Resveratrol	Diabetes patients [414]	Glucoregulation, ↑ insulin sensitivity, ↑ potency of hypoglycemic agents and antidiabetic therapies
	Obeses patients [414]	↑ or↓ insulin sensitivity ↓ adipocyte size ↓ or no effect on circulating inflammatory cytokines ↑ adiponectin
	Overweight and obese adolescents [425]	↓ insulin resistance ↓ non-alcoholic fatty liver disease (NAFLD)
	NAFLD patients [426]	**no effect on anthropomorphic** measurements, insulin markers, lipids profile, blood pressure ↓ NAFLD ↓ALT
	Cardiovascular diseases [414]	↓ or no effect on plasma lipid profile/Chol ↓ systolic blood pressure ↑ Flow-mediated dilatation ↓ pulse-wave velocity
**Whole polyphenols diets/foods**		
Apple	Middle-age women [419] Men and women [418]	↓ risk T2D ↓ risk T2D
Berry	Men and women [418]	↓ risk T2D
Blueberry	T1D children [308]	↓ HbA1c, ↑C-peptide, ↑ erythrocyte SOD
	T2D patients [427]	↓FBG, ↓ LDL, ↓ CRP ↓ AST, ↓AST, ↓GGT
Cinnamon	T2D patients [428]	↓ CV risk, ↑ insulin sensitivity
Curcumin	Diabetic patients [308]	Improve microangiopathy
	Healthy people [429]	↑ HDL, ↓ cholesterol, ↓ lipids peroxidation
Coffee	Metabolic syndrome [430]	↓ risk T2D
Cocoa drink	Hypertensive patients [402]	**no effect on insulin resistance** **no effect on blood pressure**
Dark chocolate	Healthy people [400] and Hypertensive patients [398] Healthy people [400]	↑ insulin sensitivity, ↓ blood pressure ↑ QUICKY (insulin sensitivity) ↓ HOMA-IR
Whole Grains rich diet	Obesity and T2D patients [431]	↓ risk T2D
Grape seed extract	T2D patients [432]	↓ glycaemia, ↓ inflammation **no effect on HOMA-IR**
Krill oil (rich inѠ-3)	T2D patients [409]	↓ blood C-peptide levels, ↓ HOMA-IR, ↑ HDL
Purple grape juice	Coronaries patients [393]	↓ ox LDL
Strawberry	Obese patients [405]	↓ risk factors for CVD and stroke ↓ diabetes
Tea	Middle-age women [419] Meta-analysis [433] Non obese people [434]	↓ risk T2D Prevention of T2D development ↓ risk of obesity, ↓ FBG
Green tea	T2D patients [435] Borderline diabetes or diabetes [408]	↑ levels of insulin ↓ body weight and BMI ↓ blood pressure, ↓blood glucose ↓ HbA1c, ↓HOMA index
RWPs – french Corbières AOC	Healthy people [19,20]	↓ weight, ↓ glycaemia Hypoglycemia effect

AST, AST, GGT: transaminases; BMI: body mass index; CVD: cardiovascular disease; CRP: C-reactive protein; DBP: diastolic blood pressure; FBG: fasting blood glucose; HOMA-IR: insulin resistance index; NAFLD: non-alcoholic fatty liver disease.

**Table 7:** Beneficial effects of several polyphenol-rich natural sources on in vitro and in vivo models of diabetes.

Polyphenols	Experimental Models	Efficacy
Curcumin	T2D-rats [436]	ROS scavenger ↓ nephropathy
	STZ-induced diabetic rats [437]	Protect endothelial dysfunction in the iris : ↓ retinopathy
	STZ-induced diabetic rats [438]	Improves mesenteric arteriolar function ↓ ROS artery, ↓ PKC-βII ↓ glycemia
	db/db mice [439]	↓ glycemia, ↓ weight
	Ob/ob mice [440]	↑ glycemic control, ↑insulin sensitivity, ↑ leptin/adiponectin
	Bovine aorta [441]	↓ lipid peroxidation, ROS scavenger
Tea Flavonoids	RINm5f (β-cells) [375]	ROS scavenger Fer and iron scavenger ↓ ROS production
Tea EGCG	RINm5f (β-cells) [442]	↑ mitochondrial activityprotect against oxidative stress ↑ SOD activity ↓ ROS production, ↓ caspase 8
	ex vivo skin [443,444]	protection against UV ↑ GSH, ↑ GPx activity
	in vitro [445]	prevention of hyperglycemia ↑ insulin activity protection of β cells
	STZ-induced diabetes in rats [446]	↓ β cells lost
	(OB/OB) mice [447]	↓ hepatic steatosis ↓ injury in obese mice
	(OB/OB) mice [448]	↓ intestinal lipid absorption, ↓ body mass, ↓ lipid accumulation in liver and adipocyte, ↑ insulin sensitivity, ↑ TAOC
α lipoic acid	STZ-induced diabetes in rats [449]	↓ FBG, ↓ HbA1c improve dyslipidemia ↑ SOD activity, ↑endogenous Vit C ↓ MDA and 4-HNE in aorta ↓ DNA damages good vascular morphology
Procyanidin B2 (grape seed)	STZ-induced diabetes in rats [450] Β-cells	↓ plasma glucose Insulin mimetic effect
Resveratrol	Zucker fatty (ZF) rats [451] (Obese and T2D)	↓ T-Chol, ↓ TG
	STZ-induced T2 diabetes in rats [452]	delay insulin resistance ↓ insulin secretion (hyperinsulinemia)
	Endothelial cells of rats [453]	↓ ROS, ↓NADPH oxidase, ↓inflammation ↓ LDL, antioxidant activity
RWPs extract Provinols^TM^	Zucker fatty (ZF) rats : Obese and T2D [242]	Improve glucose metabolism ↓ plasma glucose, ↓ fructosamine ↓ TG, ↓T-Chol, ↓ LDL Improve cardiac performance (↗ left ventricular and cardiac input) ↓ peripheral arteriole resistances Corrected endothelial dysfunction : in aorta : ↑ NO availability, ↑ NO, ↑ eNOS activity, ↓ O_2_, ↓ NADPH ox in mesenteric artery : ↑ EDHF
RWPs – french Corbières AOC	STZ-induced diabetes in rats and Fructose diet [19,20]	↓ weight, ↓ glycemia ↓ plasma glucose ↓ plasma lipids
	RINm5f (β-cells) [442]	↑ mitochondrial activity protect against oxidative stress ↑ SOD activity ↓ ROS production, ↓ caspase 8
SOD/CAT mimetics	animal models of diabetic neuropathy [263,264,265]	improve neuropathy
translocase of inner mitochondrial membrane	Mice [263]	improve nephropathy
tempol	Mice SOD-knockout [264]	improve nephropathy
overexpression of MnSOD	Mice [262]	improve retinopathy

EDHF: endothelium derived hyperpolarizing factor; FBG: fasting blood glucose; MDA and 4-HNE: lipids peroxide; NO: nitric oxide; ROS: reactive oxygen species; SOD: superoxide dismutase; TG: triglycerids.
